# Characterizing the Microbiome and Prevalence of *Wolbachia* in *Culex* *pipiens* Complex and *Culex* *restuans* Mosquitoes in the Midwest United States

**DOI:** 10.1007/s00248-026-02750-1

**Published:** 2026-04-01

**Authors:** Rebecca E. Cloud, Patrick Irwin, Ephantus J. Muturi, Carla E. Cáceres

**Affiliations:** 1 Program in Ecology, Evolution & Conservation Biology, University of Illinois Urbana-Champaign, 433 Morrill Hall, 505 S. Goodwin Ave, Urbana, IL 61801 USA; 2 Department of Entomology, University of Wisconsin-Madison, Madison, WI USA; 3 Northwest Mosquito Abatement, Wheeling, IL USA; 4 Crop Bioprotection Research Unit, National Center for Agricultural Utilization Research, Agricultural Research Service, United States Department of Agriculture, Peoria, IL USA; 5 Department of Evolution, Ecology, and Behavior, School of Integrative Biology, University of Illinois Urbana-Champaign, Urbana, IL USA

**Keywords:** *Culex pipiens* complex, *Culex restuans*, *Wolbachia*, Microbiome, Spatiotemporal

## Abstract

Despite the ecological importance of symbiotic relationships, few studies have explored how microbial communities vary across species complexes or within hybrid zones. We characterized the spatial and temporal variation in microbial communities in *Culex pipiens* complex and *Culex restuans* mosquitoes, the most important vectors for West Nile virus in the midwestern United States. *Cx. pipiens* complex and *Cx. restuans* mosquitoes were collected monthly from May to September 2023 from three geographic regions (Champaign County, IL, Cook County, IL and Dane County, WI). DNA from individual mosquitoes was sequenced using Illumina NovaSeq amplicon sequencing to identify the mosquito species and characterize their bacterial communities. Microbial composition differed between *Cx. pipiens* complex and *Cx. restuans*, driven by high relative abundance of *Wolbachia* in *Cx. pipiens* complex compared to *Cx. restuans*, which also contributed to greater within-species microbiome variability. Despite the limited sample sizes for some forms and hybrids, there were no detectable differences in the bacterial diversity or community composition among the five observed *Cx. pipiens* forms. Microbial diversity also varied regionally and over the sampling season, increasing later in the season. These findings demonstrate a microbial convergence within *Cx. pipiens* complex and reveal that spatiotemporal factors influence acquisition of environmentally acquired microbes highlighting the dynamic nature of mosquito-microbe interactions.

**Supplementary Information:**

The online version contains supplementary material available at 10.1007/s00248-026-02750-1.

## Introduction

Interactions between microbes and their hosts have emerged as key mechanisms driving evolutionary and ecological processes across multiple taxa. Microbes can shape host phenotype and impact host fitness through nutrient acquisition [1], resistance to pathogens [2], and altering reproductive strategies [3]. Moreover, the influence of symbioses can vary across space and time as environmental factors, migration, and seasonal shifts shape microbial communities, leading to spatiotemporal variation in host fitness [4].

Studying microbial diversity within species complexes and in hybrid zones presents an excellent opportunity for understanding the relative contributions of genetics and the environment on the microbiome in a natural setting. Phylosymbiosis has been proposed to explain the pattern in which closely related organisms share more similar microbial communities [5], though counterexamples exist, attributing most microbial variation in hosts to the environment [6]. However, as environmental and land-use change redistributes spatial and temporal habitats of insect populations, hybrid zones are shifting as well [7]. If hybrid forms are morphologically indistinguishable from parental forms, this shift in the hybrid zone becomes even more difficult to characterize. Thus, exploring how spatiotemporal and genetic variation can synergistically influence trends in microbial diversity within hybrid complexes is becoming increasingly important.

Among host-associated microbes, endosymbionts have intimate relationships with their hosts, influencing traits through ecological interactions commonly perpetuated by vertical inheritance. The extent to which maternally inherited endosymbionts interact with and shape environmentally acquired microbial communities is an active area of research. For example, the intracellular endosymbiont *Wolbachia* found in over 50% of all arthropods can facilitate, suppress, or be suppressed by various microbes, altering the overall microbial diversity and communities present within hosts, although effects are dependent on host genotype and environment [8, 9]. Further, *Wolbachia* has famously been used as a biocontrol strategy in mosquitoes due to reproductive manipulations of its hosts and influence on pathogen dynamics of several mosquito-borne diseases via pathogen blocking, though effects vary [10, 11]. Understanding the intricate interplay between the host microbiome, vertically transmitted endosymbionts, and host, including population and community-scale interactions such as disease transmission, is essential for unraveling the complexities of vector ecology.

*Culex pipiens*, one of the primary vectors of West Nile virus (WNV) in the United States [12], exists as a species complex which includes forms *Cx. pipiens* f. *pipiens*,* Cx. pipiens* f. *molestus*, and *Cx. quinquefasciatus*. All forms readily hybridize with one another where their ranges overlap [13, 14]. *Culex restuans*, another vector of WNV in the U.S., is often studied with *Cx. pipiens* due to overlap in range and morphological resemblance [15]. Apart from temporal and behavioral differences between the two *Culex* species, the microbiomes of *Cx. restuans* and *Cx. pipiens* mosquitoes have been shown to vary significantly in diversity and in abundance of *Wolbachia* [16, 17]. However, microbial differences, including differences in *Wolbachia* prevalence, among *Cx. pipiens* forms and hybrids have yet to be explored. In addition, few studies have explored how *Culex* microbiomes vary across species and through space and time and how this variation corresponds with *Wolbachia* prevalence among populations [18, 19].

To disentangle the interactions between mosquito hosts, the microbiome, and *Wolbachia*, we characterized spatiotemporal variation in microbial diversity, community structure, and *Wolbachia* prevalence in *Cx. restuans* and *Cx. pipiens* complex mosquitoes. Our goals were to: (1) describe the assemblage structure of *Cx. pipiens* complex across a latitudinal gradient and over a sampling season in the midwestern United States, (2) assess the relative contributions of region, sampling month, and *Culex* species/form to variations in microbial composition and diversity, and (3) determine the prevalence of *Wolbachia* in *Cx. pipiens* and *Cx. restuans* mosquitoes. Determining how genetic and environmental factors influence microbial composition and diversity can provide insights into potential changes in host fitness within hybrid zones across spatiotemporal gradients.

## Methods

### Sample Collections

To determine the spatial and temporal distribution of *Cx. pipiens* and *Cx. restuans* along a latitudinal gradient from Madison, Wisconsin to Champaign, Illinois we sampled adult mosquitoes monthly between May - September 2023 using CDC gravid traps from five sites in each of three regions: Dane County, WI; Cook County, IL; and Champaign County, IL (Table S1). Average temperature across the sampling period May - September 2023 in Dane County, WI was 19.7 °C with a 2023-year average of 9.9 °C and an average precipitation of 77.19 cm. In Cook County, IL, average temperature over the sampling period was 20.3 °C with an average temperature for 2023 of 11.5 °C and average precipitation of 92.35 cm. In Champaign County, IL, the sampling period average was 21.1 °C with a 2023-year average of 12.3 °C and 81.23 cm [20].

Gravid traps were operated for 20 to 24 h. Mosquito specimens were euthanized on dry ice immediately upon collection and transported to the Carl R. Woese Institute for Genomic Biology at the University of Illinois Urbana-Champaign (Urbana, IL) for identification. Over the sampling period, more than 4000 mosquitoes were collected and approximately 2000 mosquitoes were identified as *Cx. restuans* or *Cx. pipiens* complex using established morphological markers [21]. A random sample of 360 mosquitoes was selected for DNA sequencing, stratified by region, month, and morphological identification to species (Table S1). Individual mosquitoes were rinsed 3 times in sterile water, placed in 70% ethanol for 10 min, rinsed 5 times in sterile water and once in sterile 0.8% saline solution [16].

### DNA Extractions, Fluidigm PCR, & Illumina Sequencing

DNA extractions of whole-body mosquitoes, quality control (QC), target amplification via Fluidigm multiplex polymerase chain reaction (PCR) (Standard Biotools), and NovaSeq 6000 (Illumina) sequencing were performed by DNA Services at the Roy J. Carver Biotechnology Center at the University of Illinois Urbana-Champaign. Briefly, mosquitoes were lysed using the PowerSoil Pro kit (Qiagen), and the remainder of the extraction was performed using the MagMax Plant DNA isolation kit in the KingFisher Apex purification system (ThermoFisher). PCR amplification, harvesting, and barcoding were performed according to Fluidigm protocols. Targets included the 16S rRNA gene V3-V4 for bacterial microbiome analysis [17, 22], the CQ11 microsatellite for *Culex* genotyping [23], and the *Wolbachia* surface protein gene *wsp* for *Wolbachia* identification [24]. All primers were synthesized by IDT Corp. (Coralville, IA) and are listed in Table S2. Products were quantified on a Qubit fluorimeter. Barcoded libraries were loaded onto the NovaSeq 6000 resulting in 250nt paired-end reads. A full description of the molecular methods can be found in the Supplementary Materials.

### Data Analysis

All sequence data were analyzed using DADA2 [25] and phyloseq [26] in RStudio version 4.5.0 [27]. Forward and reverse reads were quality filtered and trimmed, denoised, merged, filtered for chimeras, and assigned taxonomy according to the DADA2 pipeline for NovaSeq data (Table S3). Taxonomy was assigned to 16S reads using the SILVA database 138.1 [28]. Reference sequences from NCBI GenBank were used to assign taxonomy to CQ11 in April 2025. Amplicon sequence variants (ASVs) assigned to these references were categorized as *Cx. pipiens* f. *pipiens*, *Cx. pipiens* f. *molestus*, or *Cx. quinquefasciatus*, and relative allele proportions were calculated for each sample to designate hybrids (see Supplementary Materials). Since the *wsp* gene was too long to merge paired-end reads, only forward reads were used to determine presence / absence of *Wolbachia* in each sample. ASVs corresponding to mitochondria, chloroplast, and other non-target reads were removed (see Supplementary Materials). ASVs represented by a single read (singletons) were also excluded from the analysis. Samples were analyzed without rarefaction to retain all data [29].

To characterize mosquito species and form for the metadata, the CQ11 microsatellite sequences were analyzed first. Samples for which morphological and molecular identification disagreed were removed from the analysis (*n* = 38). For downstream analysis, the sole *Cx. pipiens* f. *pipiens / quinquefasciatus* hybrid was removed due to low sample size of this hybrid form.

To compare metrics of microbial alpha and beta diversity between (1) *Cx. restuans* and *Cx. pipiens* and (2) between forms of the *Cx. pipiens* complex, analyses of alpha and beta diversity metrics were performed at the species level (*Cx. restuans* vs. *Cx. pipiens* - all forms) and *Cx. pipiens* form level, meaning all metrics were tested on two separate models. Alpha diversity metrics (observed richness, Shannon, and Faith’s PD) were transformed as needed to meet ANOVA assumptions of normality and homogeneity of variance measured by a Shapiro-Wilk test and a Levene test, respectively. Observed richness and Faith’s PD were square-root transformed, and Shannon was log-transformed. ANOVAs were used to compare each transformed alpha diversity metric using fixed effects for regions, month, and species/form. Individual trap location was not included as a factor due to unbalanced sampling of sites within regions resulting from variable trap success related to weather conditions and mosquito activity. P-values were adjusted using the False Discovery Rate (FDR) correction to account for multiple hypothesis testing. Tukey post-hoc tests were used to perform pairwise comparisons between levels of each fixed effect that was significant in the ANOVA using the emmeans package in R [30]. Beta diversity was assessed using multivariate PERMANOVAs (adonis2 function with 5000 permutations, vegan package) [31] on proportionally transformed relative abundance data and visualized using non-metric multidimensional scaling (NMDS) of Weighted UniFrac distances. To assess whether PERMANOVA results were driven by differences in within-group dispersion, we tested homogeneity of multivariate dispersion for significant predictors using betadisper (5000 permutations, vegan package) [31]. Because of the unbalanced nature of the data, we did not evaluate interaction terms in the models for alpha and beta diversity. To investigate differences in relative abundances of microbial taxa between *Cx. pipiens* and *Cx. restuans*, *Cx. pipiens* forms, regions, and months, we also performed a differential abundance analysis using the ancombc2 function (Analysis of Compositions of Microbiomes with Bias Correction 2) in the ANCOMBC package [32]. Differential abundance analysis was performed at the genus level due to low overlap in ASVs between species. All ANCOM-BC models included covariates for *Culex* species, region, and month apart from the *Cx. pipiens* form comparison which was performed on the dataset without *Cx. restuans*.

Since *Wolbachia* often dominated the microbiome of our *Cx. pipiens* samples, we removed *Wolbachia* from both datasets to understand microbial diversity and community composition of environmentally acquired microbes. For the *Wolbachia*-removed analysis, 3,517 ASVs assigned to the genus *Wolbachia* were removed, and the same methods as described above were applied to both species and *Cx. pipiens* form datasets to compare metrics of alpha and beta diversity.

## Results

### *Culex* Assemblage Composition in Selected Samples

**Fig. 1:**
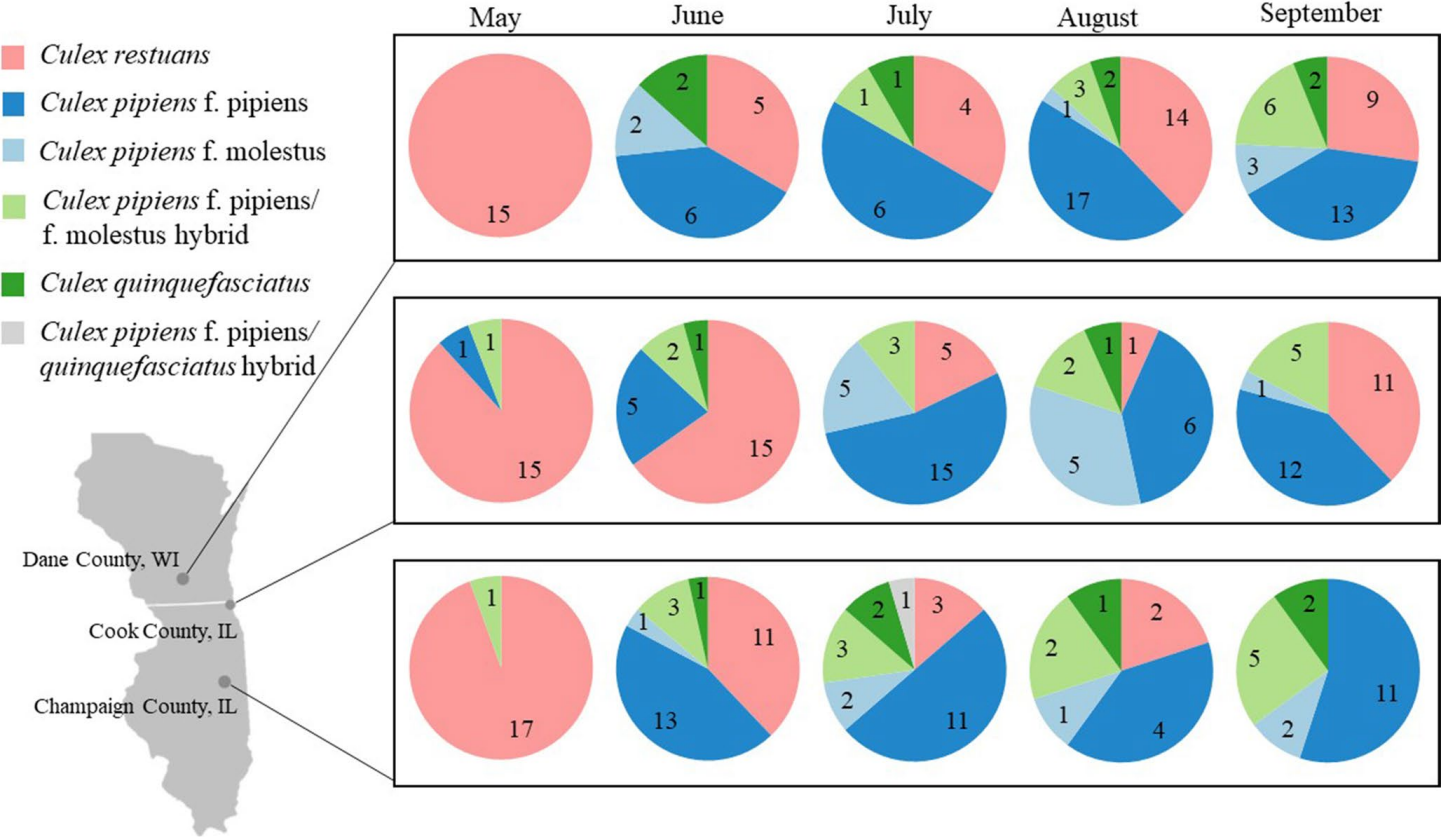
*Culex* species and form composition by region (Dane County, WI; Cook County, IL; Champaign County, IL) and month (May – September) of the subsample as determined by morphological identification and confirmed through sequencing of the CQ11 marker. Numbers correspond to counts of individual mosquitoes

The number of reads obtained per sample and information on filtering can be found in Table S3. Due to our stratified subsampling approach, the following results represent the composition of our selected subsample (*n* = 360) rather than the broader spatiotemporal distribution of *Cx. pipiens* complex populations in the study area. Of the 322 mosquitoes for which consensus was reached among morphological identification and CQ11 analysis, 120 were identified as *Cx. pipiens* f. *pipiens*, 23 as *Cx. pipiens* f. *molestus*, 36 as *Cx. pipiens* f. *pipiens /* f. *molestus* hybrids, 15 as *Cx. quinquefasciatus*, 1 as *Cx. pipiens* f. *pipiens / quinquefasciatus* hybrid, and 127 as *Cx. restuans* (Fig. 1, Table S1). Within our subsample, we observed a crossover from *Cx. restuans* to *Cx. pipiens* as the dominant species to occur in June (Fig. 1). *Cx. pipiens* f. *molestus*,* Cx. pipiens* f. *pipiens /* f. *molestus* hybrids, and *Cx. quinquefasciatus* were detected across all three regions and throughout the sampling period (June - September), though *Cx. pipiens* f. *pipiens* was consistently the most abundant form across all regions and months except May.

###  16S rRNA Alpha Diversity

**Fig. 2:**
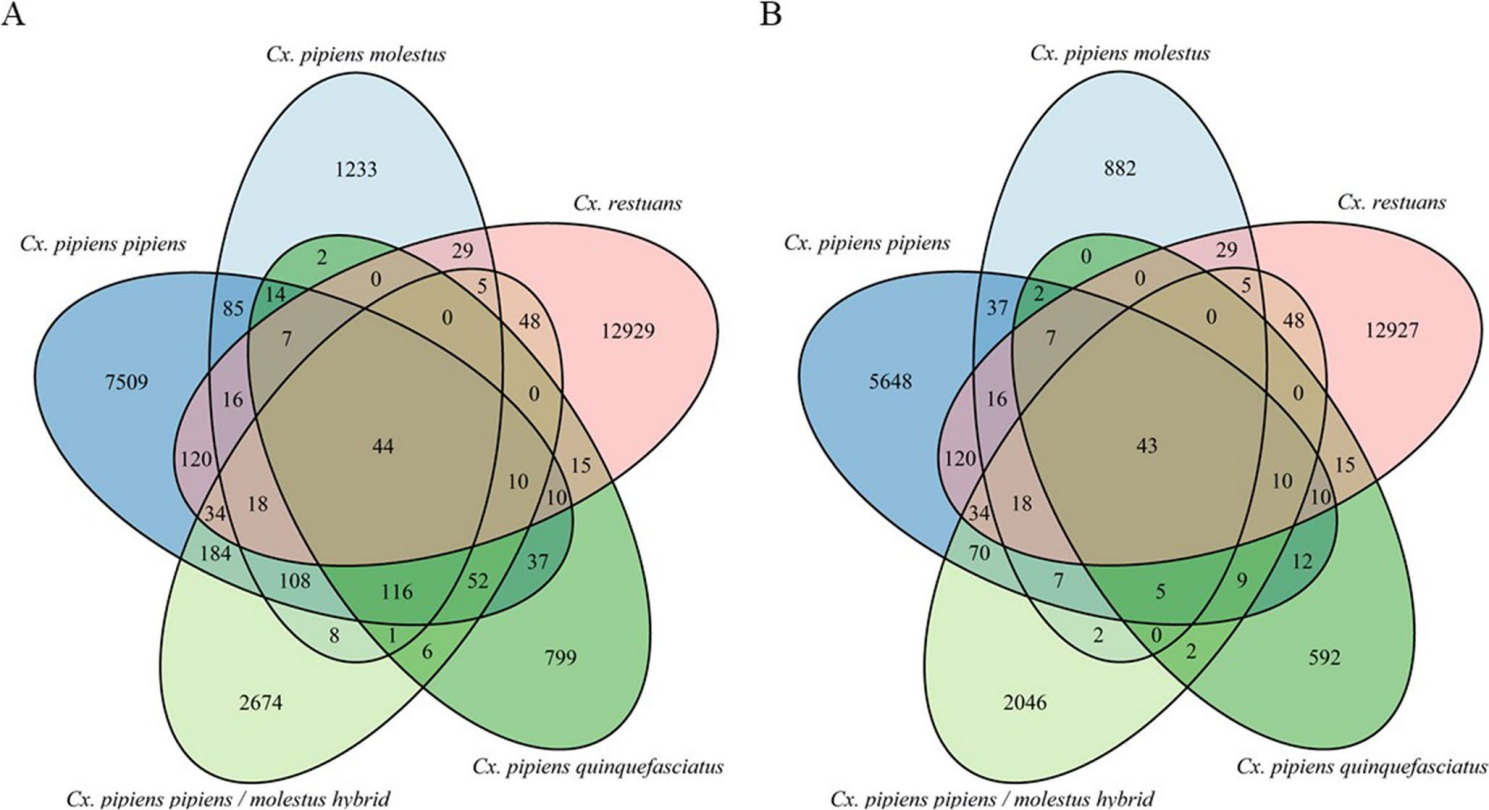
Number of shared and unique 16S rRNA amplicon sequence variants (ASVs) between *Culex* species and forms (**A**) before and (**B**) after *Wolbachia* ASVs (*n* = 3,517) were removed. The species and forms only shared 44 ASVs, one of them being a strain of *Wolbachia*. A large majority (96.29%) of ASVs were unique to a single species or form. ASVs represent 100% similarity. The number of *Wolbachia* ASVs found in each species or form can be obtained by subtracting B from A. Note that *Wolbachia* ASVs represent analytical units based on exact sequence variants and may include closely related sequences rather than distinct biological strains. Their removal was performed to control for *Wolbachia* dominance, not to assess strain diversity

The number of reads obtained per sample and information on filtering can be found in Table S3. Of the 26,113 16S rRNA ASVs, 25,144 (96.29%) were unique to a single *Culex* species or form (Fig. 2A). Only 44 (0.17%) ASVs were shared among all *Culex* species/forms (Table S4).

**Fig. 3:**
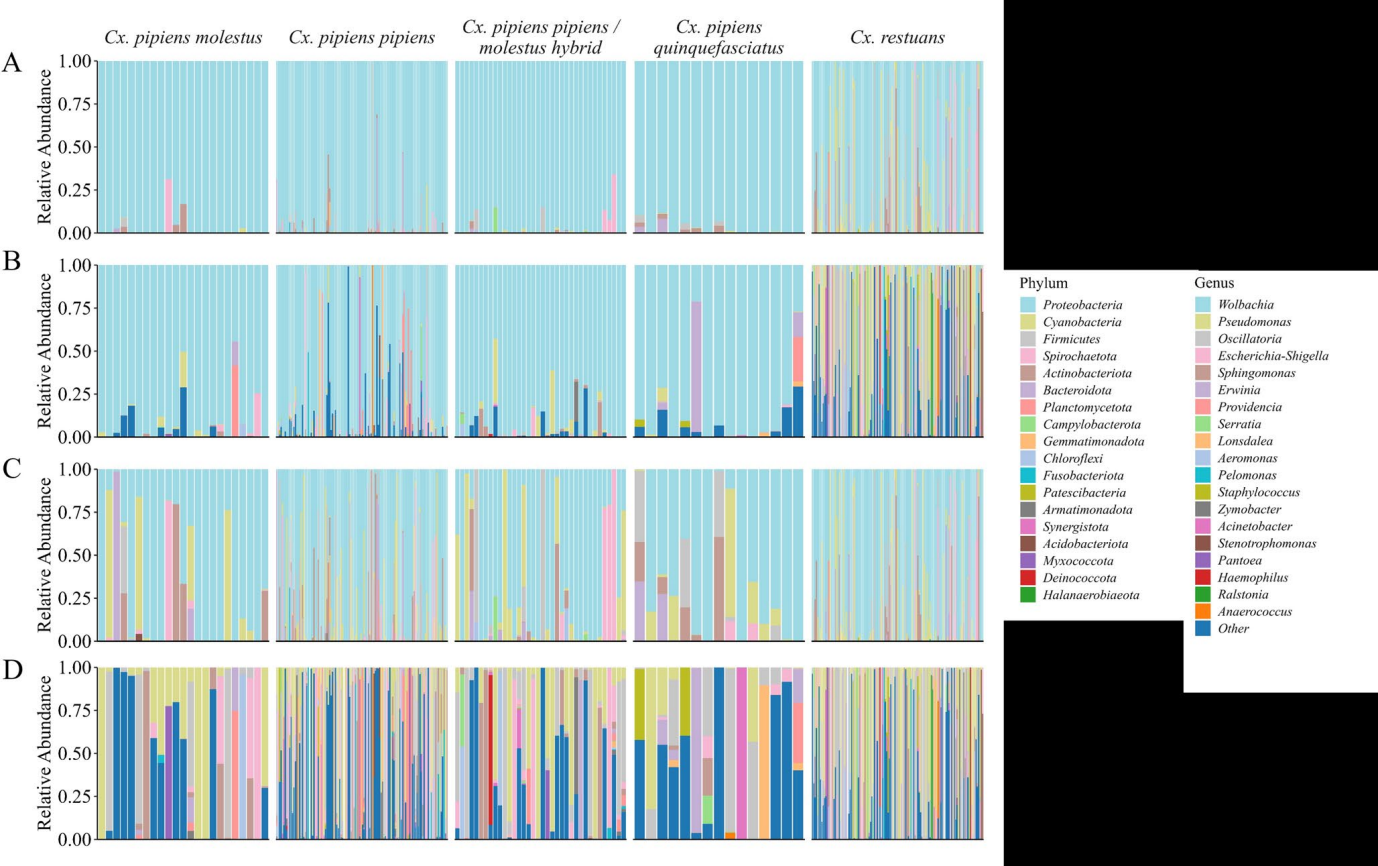
Relative abundance of the top 20 bacterial phyla (**A** & **C**) and genera (**B** & **D**), with (**A** & **B**) and without (**C **& **D**) *Wolbachia*, for each of the four *Culex pipiens *forms and *Culex restuans*. *Proteobacteria* was the most abundant phylum among both *Cx. restuans *and all *Cx. pipiens* forms. The most abundant genus for all *Cx. pipiens* forms was *Wolbachia*, while *Cx. restuans* had more even communities with *Pseudomonas *being the most abundant genus. Each bar represents the bacterial community of an individual mosquito

Relative abundances of bacterial phyla and genera across the four *Cx. pipiens* forms and *Cx. restuans* before and after removal of *Wolbachia* reads are represented in Fig. 3(A-D). The five most abundant phyla in *Cx. restuans* were *Proteobacteria* with an average relative abundance across samples of 70.88% and a 100% prevalence, followed by *Cyanobacteria* (11.54%, 81.10%), *Firmicutes* (6.44%, 39.37%), *Spirochaetota* (4.73%, 19.69%), and *Actinobacteriota* (4.11%, 23.62%) (Fig. 3A). The five most abundant genera for *Cx. restuans* were *Pseudomonas* (22.07%, 77.17%), *Oscillatoria* (17.32%, 78.74%), *Wolbachia* (7.97%, 100%), *Sphingomonas* (6.78%, 36.22%), and *Escherichia-Shigella* (5.62%, 40.16%) (Fig. 3B). For the *Cx. pipiens* complex, the five most abundant phyla were *Proteobacteria* (95.80%, 100%), *Firmicutes* (1.30%, 50.51%), *Bacteroidota* (0.90%, 23.20%), *Actinobacteriota* (0.78%, 33.51%), and *Spirochaetota* (0.63%, 50.00%) (Fig. 3A), and the most abundant genera were *Wolbachia* (84.06%, 100%), *Providencia* (2.11%, 66.49%), *Escherichia-Shigella* (1.78%, 47.42%), *Pseudomonas* (1.52%, 92.27%), and *Erwinia* (0.95%, 29.38%) (Fig. 3B). Forty-five mosquitoes (14.02%) had > 50% of their bacterial community composed of taxa other than the top 10. The “other” category differed substantially among these individuals, with some being dominated by a single rare genus, sometimes unique to that mosquito, whereas other samples had a heterogeneous mixture of rare taxa. Details on the average relative abundance and prevalence of the 40 most abundant taxa can be found in Table S5.

**Fig. 4:**
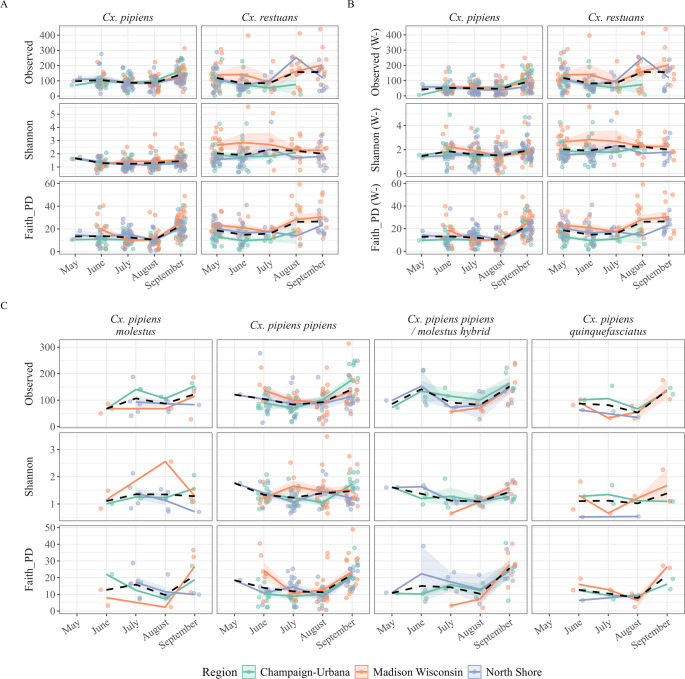
Differences in observed richness, Shannon diversity, and Faith’s phylogenetic diversity between (**A**) *Culex pipiens* and *Cx. restuans *with *Wolbachia*, (**B**) *Cx. pipiens* and *Cx. restuans* without *Wolbachia* (W-), and (**C**) four *Cx. pipiens *forms over five sampling months in three regions. While *Cx. pipiens* forms did not differ from one another in any metric with or without *Wolbachia*, *Cx. pipiens* (all forms combined) had lower estimates of Shannon and Faith’s PD than *Cx. restuans*. Although no consistent regional pattern emerged among alpha diversity metrics, estimates of observed richness and Faith’s PD were typically highest in September. Points represent observations from individual mosquitoes, shading represents standard error, and black dashed lines represent species/form averages

To compare alpha diversity between (1) *Cx. pipiens* and *Cx. restuans* and (2) the four *Cx. pipiens* forms, we investigated three alpha diversity metrics (observed richness, Shannon’s index, and Faith’s phylogenetic diversity (PD)) using multivariate ANOVAs with *Culex* species/form, region, and month as predictors (Table 1; Fig. 4). *Cx. pipiens* (all forms combined) had significantly lower estimates of Shannon diversity (F_1, 313_ = 82.26, *p* < 0.001) and Faith’s PD (F_1, 313_ = 17.93, *p* < 0.001) than *Cx. restuans*, but ASV richness did not differ significantly between the two species (Table 1). There were no significant differences in any measures of alpha diversity between the four *Cx. pipiens* forms (Table 1). Region significantly influenced all alpha diversity metrics at the species level dataset as well as Shannon diversity index for the *Cx. pipiens* complex dataset, though no generalizable regional pattern emerged. Both datasets revealed the estimates of observed richness and Faith’s PD to be highest in September (Table 1).

**Table 1:** ANOVA results from alpha diversity metrics compared between four models, (1) *Culex pipiens* and *Culex restuans* (2) the four *Cx. pipiens* forms, (3) *Culex pipiens* and *Culex restuans* with *Wolbachia* 16S rRNA ASVs removed, and (4) the four *Cx. pipiens* forms with *Wolbachia* 16S rRNA ASVs removed. For all models, observed richness and Faith’s PD were square-root transformed, and Shannon was log-transformed to fit assumptions of normality. P-values were adjusted using the False Discovery Rate (FDR) correction to account for multiple hypothesis testing

Variable		df	Res df	Observed Richness		Shannon		Faith’s PD	
				F	p	F	p	F	p
*Cx. restuans* vs. *Cx. pipiens*	Species	1	313	0.08	0.80	82.26	**< 0.001**	17.93	**< 0.001**
	Region	2	313	4.16	**0.031**	10.65	**< 0.001**	15.01	**< 0.001**
	Month	4	313	8.78	**< 0.001**	1.11	0.38	18.43	**< 0.001**
*Cx. pipiens* complex forms only	Form	3	184	1.98	0.17	1.66	0.22	1.65	0.22
	Region	2	184	2.11	0.17	3.78	**0.044**	2.12	0.17
	Month	4	184	9.94	**< 0.001**	2.58	0.064	18.39	**< 0.001**
*Cx. restuans* vs. *Cx. pipiens* (W-)	Species	1	313	55.99	**< 0.001**	12.10	**0.0013**	21.14	**< 0.001**
	Region	2	313	6.41	**0.004**	1.97	0.18	14.64	**< 0.001**
	Month	4	313	9.23	**< 0.001**	2.16	0.11	18.41	**< 0.001**
*Cx. pipiens* complex forms only (W-)	Form	3	184	1.53	0.23	0.52	0.71	1.57	0.23
	Region	2	184	4.64	**0.022**	0.014	0.99	2.08	0.17
	Month	4	184	8.80	**< 0.001**	2.57	0.064	18.16	**< 0.001**

To isolate the environmentally acquired microbes for alpha diversity analyses, we removed 3,517 ASVs assigned to the genus “*Wolbachia*” and repeated the analyses (Table 1). *Cx. pipiens* (all forms combined) still retained significantly lower estimates of Shannon (F_1, 313_ = 12.10, *p* = 0.0013) and Faith’s PD (F_1, 313_ = 21.14, *p* < 0.001) than *Cx. restuans*. Observed richness was now lower in *Cx. pipiens* than *Cx. restuans* (F_1, 313_ = 55.99, *p* < 0.001), reflecting the disproportionate reduction in *Wolbachia* ASVs from the *Cx. pipiens* complex only (Fig. 2). No significant differences were observed between *Cx. pipiens* forms after removal of *Wolbachia* (Table 1). Region no longer had a significant effect on Shannon for either dataset but was now a significant predictor of richness in the model comparing only the *Cx. pipiens* forms. The removal of *Wolbachia* did not alter the interpretation of the month’s effect on alpha diversity, and September’s richness and Faith’s PD remained the highest.

###  16S rRNA Beta Diversity

**Fig. 5:**
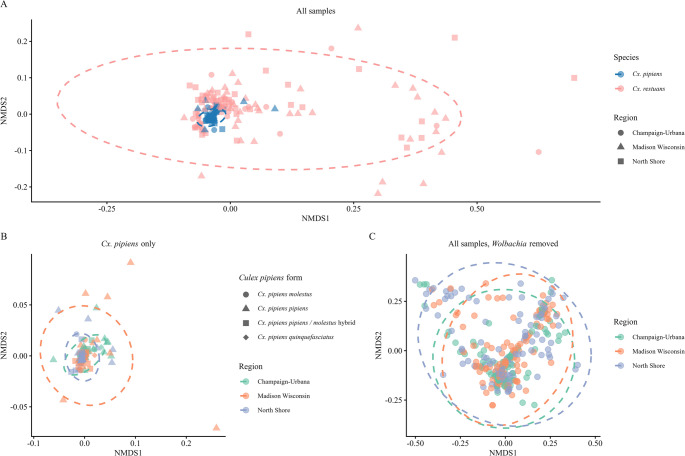
Non-metric multidimensional scaling (NMDS) based on Weighted UniFrac distances of significant PERMANOVA results for (**A**) all samples with *Cx. pipiens* forms combined, (**B**) *Cx. pipiens* forms, and (**C**) all samples with *Wolbachia* reads removed from the dataset. Microbial community composition in *Cx. restuans* was significantly different from the *Cx. pipiens* complex (all forms combined) likely due to strong differences in within-group dispersion. After *Wolbachia* was removed, no significant differences between the species remained. Instead, composition of environmentally acquired microbes differed by region alone. Region was a weak and heteroscedastic predictor, likely also influencing the significance of the analyses due to dispersion

Significant differences in beta diversity as measured by Weighted UniFrac distances were visualized in an NMDS plot (Fig. 5). A multivariate PERMANOVA (adonis2) revealed significant differences in beta diversity between species (R² = 0.128, F_1, 313_ = 50.58, *p* < 0.001) and regions (R² = 0.017, F_2, 310_ = 3.28, *p* = 0.003), but not months (R² = 0.015, F_4, 310_ = 1.43, *p* = 0.09) (Fig. 5A). However, beta dispersion of species (F_1, 319_ = 342.06, *p* < 0.001) and region (F_2, 318_ = 4.69, *p* = 0.008) were significant, indicative of heteroscedastic data. For the *Cx. pipiens* complex only dataset, region was a significant but weak predictor of community composition (R² = 0.032, F_2, 184_ = 3.18, *p* = 0.004), but month (R² = 0.030, F_4, 184_ = 1.48, *p* = 0.12) and form (R² = 0.018, F_3, 184_ = 1.19, *p* = 0.26) were not (Fig. 5B).

To further understand community composition of environmentally acquired microbes, we again removed *Wolbachia* from both datasets and repeated the analyses (Fig. 5C). The species level dataset had region (R² = 0.021, F_2, 313 =_ 3.50, *p* < 0.001) as weak significant predictor of beta diversity, whereas species (R² = 0.004, F_1, 313_ = 1.38, *p* = 0.18) and month (R² = 0.017, F_4, 313_ = 1.14, *p* = 0.066) were not. Region was heteroscedastic (F_2, 318_ = 6.98, *p* = 0.0014). Removing *Wolbachia* did not change the interpretation for the *Cx. pipiens* forms dataset.

### Differential Abundance Analysis (ANCOM-BC2)

Differential abundance analysis between the two *Culex* species revealed that *Wolbachia* and *Gibsiella* were enriched in *Cx. pipiens*, and *Cutibacterium*,* Sphingomonas*,* Escherichia-Shigella*,* Kluyvera*,* Acinetobacter*, and *Pseudomonas* were enriched in *Cx. restuans* (Table S6. There was also a greater abundance of unknown taxa in *Cx. restuans* than *Cx. pipiens.* Within the *Cx. pipiens* complex, *Erwinia* was more abundant in *Cx. pipiens quinquefasciatus* and *Cx. pipiens molestus* than in *Cx. pipiens pipiens* or *Cx. pipiens pipiens / molestus* hybrids (Table S7). Further, *Lonsdalea* was more abundant in *Cx. pipiens pipiens* and *Cx. pipiens quinquefasciatus* than in *Cx. pipiens molestus* or *Cx. pipiens pipiens / molestus* hybrids (Table S7). *Aeromonas*,* Escherichia-Shigella*,* Kluyvera*,* Erwinia*,* Coetzeea*,* Gibbsiella*,* Zymobacter*, and *Acinetobacter* were more abundant in Madison than Champaign-Urbana or North Shore (Cook County), *Cutibacterium*,* Allobacillus*,* Kluyvera*,* Erwinia*,* Providencia*,* Lonsdalea*, and *Acinetobacter* were more abundant in Champaign-Urbana than in North Shore or Madison, and *Sphingomonas*,* Pelomonas*,* Coetzeea*, and *Gibbsiella* were more abundant in North Shore than in Champaign-Urbana or Madison (Table S8). *Pelomonas* was more abundant in June than in May, and *Gibbsiella* was more abundant in May and June than in July and August (Table S9).

### *Wolbachia* Prevalence: *Wolbachia* Surface Protein (*wsp*)

Although 100% of the mosquitoes tested were found with some level of *Wolbachia* 16S rRNA ASVs, we additionally sequenced *wsp* to verify the presence of *Wolbachia* in our samples. We defined *Wolbachia* presence as detection of any 16S rRNA or *wsp* reads (> 0) without applying read count thresholds to maximize transparency and allow for broader interpretation. In the *Cx. pipiens* complex, *wsp* was detected in > 96% of the individuals (Table 2). In contrast, *wsp* was only found in 79.5% of the *Cx. restuans* individuals and with much lower read counts compared to *Cx. pipiens*.

**Table 2:** *Wolbachia* prevalence in *Culex* species/forms based on 16S rRNA and *Wolbachia* surface protein (*wsp*) read counts show competing results regarding individual level *Wolbachia* infection status. Prevalence is defined as detection of any reads (> 0) without applying read count thresholds. While 100% of samples were found with some level of *Wolbachia* 16S rRNA reads, not all mosquitoes were found with *wsp*. Read counts were also considerably lower in *Cx. restuans* than the *Cx. pipiens* complex forms, and the biological significance of low read counts in *Cx. restuans* remains uncertain (see Discussion). Read counts are based on post-filtration numbers. Mean and standard error are based on non-zero counts.

	*Wolbachia* 16S rRNA Reads			*Wolbachia* *wsp* Reads		
Species/Form	Prevalence (#positive, %)	Mean	Standard Error	Prevalence (#positive, %)	Mean	Standard Error
*Cx. pipiens* f. molestus	23 (100%)	569,368.5	55,862.8	22 (95.7%)	148,038.4	20,308.3
*Cx. pipiens* f. pipiens	120 (100%)	513,155.3	27,802.4	117 (97.5%)	147,573.8	9,786.4
*Cx. pipiens* f. pipiens / f. molestus hybrid	36 (100%)	573,796.5	48,379.4	36 (100%)	159,531.6	21,851.7
*Cx. pipiens quinquefasciatus*	15 (100%)	438,041.4	59,663.0	15 (100%)	105,998.1	17,697.7
*Cx. restuans*	127 (100%)	192.9	16.4	101 (79.5%)	26.3	4.1

## Discussion

Microbial symbioses are essential to understanding the biology of an organism, and bacterial endosymbionts such as *Wolbachia* have unique interactions with their arthropod hosts that impact host physiology and disease ecology. We assessed how microbial communities and *Wolbachia* infections varied spatially and temporally in *Cx. restuans* and *Cx. pipiens* complex populations. To our knowledge, this is the first study to characterize and compare microbiomes between forms of the *Culex pipiens* complex. Our findings provide valuable insights into how mosquito ecology and microbial ecology intersect across spatial and temporal scales within *Culex* species and populations.

Within our sample, we observed a crossover from *Cx. restuans* to *Cx. pipiens* around late June for all regions consistent with historical data from the Midwest [33]. This crossover has been estimated to occur earlier in the summer under warmer conditions as measured by exceeding degree days with a base of 17 °C and a daily maximum temperature threshold of 27 °C [34], and is potentially explained by the sensitivity of *Cx. restuans* to increasing temperatures [35]. We might expect slight differences in the timing of this shift across different climatic regions. However, given our stratified subsampling design and since sampling was conducted only once per month, we do not have the support needed to determine exact crossover periods.

We observed *Cx. pipiens* f. *molestus* and *Cx. pipiens* f. *pipiens -* f. *molestus* hybrids in all regions from June - September. *Cx. pipiens* f. *molestus* is adapted to urban areas [14] and many of our traps were placed in urban locations within each region. *Cx. pipiens* f. *molestus* and its hybrid forms have a higher propensity to feed on humans [13], and the hybrid forms may have a higher vector competence for WNV [36]. Thus, the existence of *molestus* ancestry and presence of hybrids in all regions is of epidemiological importance and requires more attention.

Our findings provide evidence of northward expansion of *Cx. quinquefasciatus*, which is believed to primarily occur in the southern U.S. with a hybrid zone between 39°N and 36°N [37]. Although previous studies suggest that *Cx. quinquefasciatus* cannot undergo true diapause [38], we found *Cx. quinquefasciatus* as far north as Dane County, WI. While mosquitoes of *Cx. quinquefasciatus* ancestry have previously been detected in Champaign [39, 40] and Cook counties [41], this finding contributes support for the northward expansion of *Cx. quinquefasciatus* [15].

*Culex restuans* had more diverse and different microbial communities than *Cx. pipiens*, but no differences in microbial diversity or community composition were observed among *Cx. pipiens* forms. However, significant heterogeneity of dispersion between species suggests that observed community-level differences also reflect differences in within-species variability. Muturi et al. (2016) sampled similar areas in Champaign-Urbana in 2014 and also found *Cx. restuans* to have a more even microbiome and a more variable community structure due to the large relative abundance of *Wolbachia* in *Cx. pipiens* not found in *Cx. restuans* [16]. However, removing *Wolbachia* from our beta diversity analyses of community composition changed the outcome of results; the community composition of *Cx. restuans* and *Cx. pipiens* were no longer different, but instead the microbial composition was significantly correlated with the region of collection. This may suggest that microbial community structures of sympatric *Cx. pipiens* and *Cx. restuans* are similar, but the overwhelming abundance of *Wolbachia* in *Cx. pipiens* masks spatial and temporal similarities of microbial community composition between the two mosquito species. However, our analyses did not include interaction terms due to unbalanced sample sizes, which may have limited our ability to detect form-specific or species-specific spatial and temporal shifts in the microbiome.

Temporal effects were apparent throughout our microbial data, markedly with the increase in alpha diversity in September. Seasonal increases in alpha diversity of *Cx. pipiens* and *Cx. restuans* in early autumn have been previously documented [42]. This increase could be explained by seasonal changes in nectar sources to adult mosquitoes, either due to seasonal availability or behavioral shifts in feeding preferences [43]. Host blood meal source is known to significantly alter microbial composition and diversity in mosquitoes [44]. The function of the microbiome is essential for diapause [45], and increasing microbial richness and phylogenetic breadth of the community members could be associated with building up fat stores or increasing the functional potential of the microbiome for diapause preparation. We suggest further investigation into the relationship between the microbiome and diapause as we did not have the statistical power to identify any microbial taxa that may be increasing in September. We also acknowledge that these spatiotemporal patterns should be interpreted within the context of our sampling design, which spanned a relatively narrow latitudinal gradient and a single sampling season (2023). Future studies incorporating broader geographic and temporal ranges, as well as a diversity of habitat types, would strengthen understanding of environmental factors shaping *Culex* microbiomes.

We found *Wolbachia* in all samples but was more abundant in *Cx. pipiens* compared to *Cx. restuans*. *Cx. pipiens* is known to have a high prevalence of *Wolbachia* in the United States [16, 46, 47]. While we report near fixation of *Wolbachia* in *Cx. pipiens* in the regions we sampled, we encourage investigation of *Cx. pipiens* populations at intermediate frequencies of *Wolbachia* infection as such populations provide an opportunity to explore the ecology and evolution of this unique endosymbiotic relationship. Given *Wolbachia’s* potential to constrain microbial diversity of its hosts thus limiting microbiome function [9], we expect greater intraspecific variation of host fitness in *Cx. pipiens* populations with intermediate frequencies of *Wolbachia* infection. Though *Wolbachia* transinfections and releases are becoming a popular biocontrol mechanism due to *Wolbachia’s* reproductive manipulations and pathogen blocking effects [11], less is known about the effects of native *Wolbachia* infections on vector competence of its hosts [1]. Population level surveys comparing vector competence for common arboviruses such as WNV between *Wolbachia* hosts and non-hosts would provide invaluable insight into *Wolbachia’s* effects on disease dynamics in natural systems.

In contrast to *Cx. pipiens*, the prevalence of *Wolbachia* in *Cx. restuans* is a developing area of research. Of the few papers investigating *Wolbachia* in *Cx. restuans*, *Wolbachia* seems to be prevalent but not abundant [16], or rare/absent [17, 22, 47, 48]. In addition to using 16S rRNA to evaluate *Wolbachia* prevalence and relative abundance, we also used *wsp* to confirm *Wolbachia* infection. Most of our *Cx. restuans* samples contained *wsp* reads, but read counts were far below the range for *Cx. pipiens*. While these findings could indicate biologically relevant *Wolbachia* infections, other explanations include index hopping, a multiplex sequencing artifact in which a low percentage of barcoded reads are incorrectly assigned to the wrong sample [49], or contamination, though rigorous surface sterilization was done to minimize this, and decontam analysis using negative controls identified only four contaminant ASVs (see Methods). We encourage additional surveys and recommend transparency via data accessibility to better formulate a consensus surrounding the prevalence and abundance of *Wolbachia* in *Cx. restuans*, an important early-season vector of WNV.

## Supplementary Information

Below is the link to the electronic supplementary material.

Supplementary Material 1

## Data Availability

Data generated from this research are available on the Illinois Data Bank 10.13012/B2IDB-4794358_V1.
