# Supplementary material for: Characterizing the Microbiome and Prevalence of Wolbachia in Culex pipiens Complex and Culex restuans Mosquitoes in the Midwest United States
Source: Microb Ecol. 2026 Apr 1;89:103. doi: 10.1007/s00248-026-02750-1 (PMC13167863; doi:10.1007/s00248-026-02750-1)
Supplement: Supplementary file 1 [file 248_2026_2750_MOESM1_ESM.docx]

**Supplementary Materials**

**Characterizing the microbiome and prevalence of *Wolbachia* in *Culex* *pipiens* complex and *Culex restuans* mosquitoes in the Midwest United States**

Rebecca E. Cloud^1^, Patrick Irwin^2,3^, Ephantus J. Muturi^4^, Carla. E. Cáceres^1,5^

1. Program in Ecology, Evolution & Conservation Biology, University of Illinois Urbana-Champaign, Urbana, IL, USA

2. Department of Entomology, University of Wisconsin-Madison, Madison, WI, USA

3. Northwest Mosquito Abatement, Wheeling, IL, USA

4. Crop Bioprotection Research Unit, National Center for Agricultural Utilization Research, Agricultural Research Service, United States Department of Agriculture, Peoria, IL, USA

5. Department of Evolution, Ecology, and Behavior, School of Integrative Biology, University of Illinois Urbana-Champaign, Urbana, IL, USA

* Corresponding author: Rebecca E Cloud, rcloud3@illinois.edu

**Table S1.** Trap locations, dates, and sample size. Mosquitoes selected for sequencing were (1) only those in good physical condition (2) randomly stratified by region, month, and morphological identification. “Caught” mosquitoes represent all female *Culex* spp. found in a trap regardless of physical condition. Note that two sampling events occurred in Champaign-Urbana in August due to low trap abundance on 8/15/23.

| Location Name  (Latitude, Longitude) | May (**Sampled**/ Caught) | June (**Sampled**/ Caught) | July (**Sampled**/ Caught) | August (**Sampled**/ Caught) | September (**Sampled**/ Caught) |
| --- | --- | --- | --- | --- | --- |
| **Champaign-Urbana** | | | | | |
| Southeast Urbana residence (40.092081, -88.197258) | 5/24/23 (**3**/37) | 6/23/23 (**9**/41) | 7/21/23 (**3**/47) | 8/15/23 (**0**/1) & 8/22/23 (**0**/6) | 9/12/23 (**4**/35) |
| Brownfield Woods  (40.144733, -88.163402) | 5/24/23 (**4**/53) | 6/23/23 (**9**/19) | 7/21/23 (**8**/69) | 8/15/23 (**3**/4) & 8/22/23 (**1**/3) | 9/12/23 (**3**/4) |
| West Urbana residence  (40.105822, -88.216435) | 5/24/23 (**3**/43) | 6/23/23 (**7**/33) | 7/21/23 (**4**/43) | 8/15/23 (**3**/3) & 8/22/23 (**3**/8) | 9/12/23 (**6**/48) |
| Trelease Woods (40.129625, -88.143619) | 5/24/23 (**7**/30) | 6/23/23 (**3**/17) | 7/21/23 (**4**/27) | 8/15/23 (0/1) & 8/22/23 (**1**/3) | 9/12/23 (**5**/26) |
| South Urbana agricultural wetland  (40.073908, -88.219594) | 5/24/23 (**6**/62) | 6/23/23 (**9**/38) | 7/21/23 (**4**/259) | 8/15/23 (0/0) & 8/22/23 (**2**/17) | 9/12/23 (**4**/675) |
| Champaign-Urbana Total (**118/**1652) | **23**/225 | **37**/148 | **23**/445 | **13/**46 | **22**/788 |
| **North Shore** | | | | | |
| Watersmeet Woods  (42.092578, -87.770011) | 5/31/23 (**3**/70) | 6/28/23 (**8**/27) | 7/25/23 (**10**/154) | 8/17/23 (**2**/15) | 9/9/23 (**4**/10) |
| Northeast Glenview residence/golf course  (42.095761, -87.801903) | 5/31/23 (**4**/84) | 6/28/23 (**3**/24) | 7/25/23 (**4**/92) | 8/17/23 (**1**/1) | 9/9/23 (**5**/8) |
| South Glenview residence  (42.061892, -87.807844) | 5/31/23 (**8**/106) | 6/28/23 (**8**/37) | 7/25/23 (**5**/409) | 8/17/23 (**7**/16) | 9/9/23 (**11**/32) |
| Glenview Lake  (42.095498, -87.815905) | 5/31/23 (**3**/21) | 6/28/23 (**0**/0) | 7/25/23 (**5**/225) | 8/17/23 (**7**/8) | 9/9/23 (**4**/33) |
| Secret Hollow Park  (42.079893, -87.803254) | 5/31/23 (**4**/39) | 6/28/23 (**4**/10) | 7/25/23 (**5**/193) | 8/17/23 (**0**/2) | 9/9/23 (**10**/12) |
| North Shore Total (**125/**1636) | **22**/320 | **23**/98 | **29**/1073 | **17**/42 | **34**/95 |
| **Madison-Wisconsin** | | | | | |
| Pheasant Branch greenway  (43.063698, -89.485982) | 5/27/23 (**0**/0) | 6/25/23 (**4**/17) | 7/23/23 (**1**/4) | 8/13/23 (**7**/100) | 9/10/23 (**3**/6) |
| Haem Park Kettle Pond  (43.06595, -89.517329) | 5/27/23 (**0**/0) | 6/25/23 (**6**/21) | 7/23/23 (**4**/11) | 8/13/23 (**7**/143) | 9/10/23 (**7**/36) |
| Sauk Creek greenway  (43.075325, -89.510113) | 5/27/23 (**15**/33) | 6/25/23 (**5**/12) | 7/23/23 (**1**/6) | 8/13/23 (**7**/91) | 9/10/23 (**0**/6) |
| Stonefield greenway  (43.082514, -89.500759) | 5/27/23 (**0**/0) | 6/25/23 (**0**/0) | 7/23/23 (**3**/7) | 8/13/23 (**1**/1) | 9/10/23 (**6**/22) |
| Pheasant Branch greenway  (43.08573, -89.481691) | 5/27/23 (**1**/1) | 6/25/23 (**0**/0) | 7/23/23 (**3**/6) | 8/13/23 (**17**/238) | 9/10/23 (**19**/152) |
| Madison-Wisconsin Total (**117/**913) | **16**/34 | **15**/50 | **12**/34 | **39**/573 | **35**/222 |

**DNA extraction**

DNA extraction, quality control (QC), amplification of targets with the Fluidigm system (Standard Biotools), and sequencing on the NovaSeq 6000 (Illumina) were conducted at the Roy J. Carver Biotechnology Center at the University of Illinois at Urbana-Champaign.

DNA extraction from mosquitoes was performed using the PowerSoil Pro kit (Qiagen, cat #47014) and the MagMax Plant DNA isolation kit (ThermoFisher, cat #A32549). Individual mosquitoes in 1.5 ml tubes were rinsed with 200 µl of PBS, and the solution was removed. The tubes were placed in a metal rack submerged in liquid nitrogen, and the mosquitoes were ground with a plastic blue pestle. Subsequently, 200 µl of solution CD1 with 10 µl of RNAseA from the PowerSoil Pro kit were added to the ground samples. The samples were kept at 4°C for 2 hours, centrifuged at 15,000g for 1 minute, and the supernatant was transferred to a new 1.5 ml tube. Then, 50 µl of CD2 solution from the PowerSoil Pro kit were added to the supernatant, vortexed for 1 minute, and centrifuged at 15,000g for 1 minute.

Next, 150 µl of the lysate from each mosquito was transferred to a well in a 96-well plate, and the remainder of the DNA extraction was completed in the KingFisher Apex instrument (ThermoFisher). Each well received 150 µl of lysate, 150 µl of 100% ethanol, and 10 µl of beads from the MagMax kit. The beads were washed with 700 µl of Wash Buffer 1, followed by another wash with 700 µl of Wash Buffer 2, both from the MagMax kit. DNA was eluted from the washed beads with 60 µl of 10 mM Tris.

The eluted DNAs were quantified using the Qubit high-sensitivity assay (ThermoFisher). A subset of the samples was run on a 1% agarose Ex-gel (ThermoFisher) to confirm DNA integrity.

**Target amplification with the Fluidigm System**

**Table S2.** List of amplicon targets, forward and reverse primer names, and primer sequences. All primers were synthesized by IDT Corp. (Coralville, IA).

| **Primer target** | **Primer name** | **Locus-specific primer sequence** |
| --- | --- | --- |
| V3_V4_F357_R805 | V3_F357_N | 5'-CCTACGGGNGGCWGCAG |
|  | V4_R805 | 5'-GACTACHVGGGTATCTAATCC |
| *wsp* | wspF | 5'-TGGTCCAATAAGTGATGAAGAAA |
|  | wspR | 5'-AAAAATTAAACGCTACTCCA |
| ank2A | ank2F | 5'-CTTCTTCTGTGAGTGTACGT |
|  | ank2RA | 5'-TCCATATCGATCTACTGCGT |
| CQ11 | CQ11F | 5'-GATCCTAGCAAGCGAGAAC |
|  | CQ11R | 5'-GAGCGGCCAAATATTGAGAC |
| ITS7_ITS4 | ITS7 | 5'-GTGARTCATCGAATCTTTG |
|  | ITS4 | 5'-TCCTCCGCTTATTGATATGC |

**Fluidigm 2 Step Access Array Amplification**

Prior to amplification all DNA samples were measured on a Qubit (Life Technologies) using the High Sensitivity DNA Kit. Samples were diluted to 2 ng/ul concentrations. A mastermix for amplification was prepared using the Roche High Fidelity Fast Start Kit and 20x Access Array loading reagent according to Fluidigm protocols. For each sample the following reagents were combined: 0.5 ul 10X FastStart Reaction Buffer without MgCl2, 0.9 ul 25 mM MgCl2, 0.25 ul – DMSO, 0.1 ul 10 mM PCR grade Nucleotide Mix, 0.05 ul 5 U/ul FastStart High Fidelity Enzyme Blend, 0.25 ul 20X Access Array Loading Reagent, and 1.95 ul water.

Mastermix was aliquoted to 48 wells of a PCR plate. To each well, 1 ul DNA sample was added. In a separate plate, primer pairs were prepared and aliquoted. 20X primer solutions were prepared by adding 2 ul of each forward and reverse primer (50 uM each), 5 ul of 20X Access Array Loading Reagent and water to a final volume of 100 ul.

4 ul of sample was loaded in the sample inlets and 4 ul of primer loaded in primer inlets of a previously primed Fluidigm LP48.48 IFC. The IFC was placed in a Fluidigm Juno for loading, PCR amplification and harvesting using the following PCR conditions: 50 °C for 2 min, 70 °C for 20 min, 95 °C for 10 min; 95 °C for 15 s, 55 °C for 30 s, followed by 10 cycles of 72 °C for 1 min; 95 °C for 15 s, 80 °C for 30 s, 60 °C for 30 s, 2 cycles of 72 °C for 1 min; 95 °C for 15 s, 55 °C for 30 s, then 8 cycles of 72 °C for 1 min; 95 °C for 15 s, 80 °C for 30 s, 60 °C for 30 s, then 2 cycles of 72 °C for 1 min; 95 °C for 15 s, 55 °C for 30 s, 8 cycles of 72 °C for 1 min; 95 °C for 15 s, 80 °C for 30 s, 60 °C for 30 s, and finally 5 cycles of 72 °C for 1 min.

Harvested product was then transferred to a new 96 well plate and diluted 1:100 in water. 1 ul of diluted product was used for a second round of amplification with Illumina linkers and barcodes.

For second round PCR the following reagents were combined: 2.0 ul 10X FastStart Reaction Buffer without MgCl2, 3.6 ul 25 mM MgCl2, 1.0 ul – DMSO, 0.4 ul 10 mM PCR grade Nucleotide Mix, 0.2 ul 5 U/ul FastStart High Fidelity Enzyme Blend, and 7.8 ul water. 15 ul of reagent mix was combined with 1 ul diluted first round PCR product and 4 ul of Illumina linker barcodes.

The PCR cycling program consisted of: 1 cycle of 95 °C for 10 min, 95 °C for 15 s, 60 °C 30 s, 14 cycles of 72 °C for 1 min, and a 3 min extension at 72 °C.

Products were quantified on a Qubit fluorimeter and stored at -20C. All samples were run on a Fragment Analyzer (Agilent Technologies) and amplicon regions and expected sizes confirmed. Samples were then pooled in equal amounts according to product concentration. The pooled products were then size selected on a 2% agarose E-gel (Life Technologies) and extracted from the isolated gel slice with Qiagen gel extraction kit (Qiagen). Cleaned size selected products were run on an Agilent Bioanalyzer to confirm appropriate profile and determination of average size.

**Sequencing on a NovaSeq 6000.**

The pool of individual barcoded libraries was denatured and spiked with 20% non-indexed PhiX control library provided by Illumina. The pool was loaded onto 1 lane of a 2-lane NovaSeq SP flowcell at a concentration of 1nM for sequencing on the NovaSeq 6000 with version 1.5 sequencing reagents. The PhiX control library provides a balanced genome for calculation of matrix, phasing and prephasing, which are essential for accurate basecalling. The libraries were sequenced from both ends of the molecules to a total read length of 250nt from each end.

The run generated .bcl files which were converted into demultiplexed compressed fastq files using bcl2fastq 2.20 (Illumina).

**Table S3.** Read tracking and ASVs numbers before downstream filtration steps.

| **Target Gene** | **Input Reads** | **Filtered Reads** | **Denoised Fs** | **Denoised Rs** | **Merged** | **Chimera Removed** | **# ASVs** |
| --- | --- | --- | --- | --- | --- | --- | --- |
| CQ11 | 62,350,560 | 56,533,105 | 56,464,058 | 56,469,555 | 53,449,339 | 52,850,171 | 3,037 |
| 16S rRNA | 233,047,285 | 208,291,515 | 207,774,385 | 207,754,334 | 197,692,417 | 193,267,305 | 32,845 |
| *wsp* | 37,817,075 | 34,645,965 | 34,572,866 | NA | NA | 34,462,362 | 2,135 |

**CQ11 Taxonomy Assignment and Filtration**

All 3,037 ASVs were assigned taxonomy using the NCBI nBLAST database. 144 ASVs had no matches, and an additional 437 non-target ASVs were removed using the phrases “PREDICTED” “Uncultured” “uncharacterized” “chloroplast” “mRNA” “Wolbachia” “chromosome” “mitochondria” “RNA” “Mutant” “virus” “phage” “clone” and “genomic”. This left 2456 ASVs which matched to 16 published *Culex* CQ11 microsatellite sequences (AY962870.1, DQ470140.1, DQ470142.1, DQ470146.1, DQ470147.1, DQ470148.1, DQ470149.1, DQ470150.1, DQ470151.1, DQ470152.1, DQ470153.1, KP675978.1, KY744217.1, KY744218.1, KY744221.1, and OL342322.1). Based on the genetic information reported in these 16 sequences to the NCBI database, CQ11 alleles were categorized into “*Culex pipiens* f. pipiens”, “*Culex pipiens* f. molestus” or “*Culex quinquefasciatus*”. The relative proportion of CQ11 alleles was calculated for each sample. Samples possessing a 20% or greater abundance of the minor allele were marked as heterozygotes/hybrids for those alleles. Samples with below 500 total reads were identified as *Cx. restuans*. Of the 360 original samples, 38 samples had incongruency between morphological and molecular identification and were removed from the dataset. Downstream analyses were performed on the 322 samples for which morphological and molecular consensus with CQ11 was reached.

**16S rRNA Filtration**

After taxonomic assignment of the 32,845 16S rRNA ASVs, we removed 665 “NA” ASVs, 45 ASVs assigned *Eukaryota* or *Archaea* at the kingdom level, 2,982 ASVs assigned to mitochondria, 151 ASVs assigned to chloroplasts, 73 singletons, and 4 ASVs identified as contaminants via six negative controls. After removing additional ASVs only found within samples that were removed due to identification mismatch, or in the controls, 26,113 ASVs remained.

**Supplementary Results**

**Table S4.** Taxonomy of the 44 ASVs shared between *Culex restuans* and four *Cx. pipiens* forms.

| Kingdom | Phylum | Class | Order | Family | Genus | Count ASVs |
| --- | --- | --- | --- | --- | --- | --- |
| *Bacteria* | *Actinobacteriota* | *Actinobacteria* | *Propionibacteriales* | *Propionibacteriaceae* | *Cutibacterium* | 1 |
|  |  |  | *Micrococcales* | *Microbacteriaceae* | *Pseudoclavibacter* | 1 |
|  | *Bacteroidota* | *Bacteroidia* | *Flavobacteriales* | *Flavobacteriaceae* | *Imtechella* | 1 |
|  | *Firmicutes* | *Bacilli* | *Staphylococcales* | *Staphylococcaceae* | *Staphylococcus* | 1 |
|  | *Proteobacteria* | *Alphaproteobacteria* | *Acetobacterales* | *Acetobacteraceae* | NA | 1 |
|  |  |  | *Caulobacterales* | *Caulobacteraceae* | NA | 1 |
|  |  |  | *Rickettsiales* | *Anaplasmataceae* | *Wolbachia* | 1 |
|  |  |  | *Sphingomonadales* | *Sphingomonadaceae* | *Sphingomonas* | 1 |
|  |  | *Gammaproteobacteria* | *Burkholderiales* | *Comamonadaceae* | *Pelomonas* | 1 |
|  |  |  | *Enterobacterales* | *Aeromonadaceae* | *Aeromonas* | 1 |
|  |  |  |  | *Enterobacteriaceae* | *Escherichia-Shigella* | 1 |
|  |  |  |  |  | *Kluyvera* | 1 |
|  |  |  |  |  | NA | 3 |
|  |  |  |  | *Erwiniaceae* | *Erwinia* | 4 |
|  |  |  |  |  | *Pantoea* | 2 |
|  |  |  |  |  | *Phaseolibacter* | 2 |
|  |  |  |  |  | *Siccibacter* | 1 |
|  |  |  |  | *Morganellaceae* | *Providencia* | 2 |
|  |  |  |  | *Pectobacteriaceae* | *Lonsdalea* | 1 |
|  |  |  |  | *Thorselliaceae* | *Coetzeea* | 1 |
|  |  |  |  | *Yersiniaceae* | *Gibbsiella* | 2 |
|  |  |  |  |  | *Serratia* | 4 |
|  |  |  |  | NA | NA | 3 |
|  |  |  | *Pseudomonadales* | *Halomonadaceae* | *Zymobacter* | 1 |
|  |  |  |  | *Moraxellaceae* | *Acinetobacter* | 1 |
|  |  |  |  | *Pseudomonadaceae* | *Pseudomonas* | 1 |
|  | *Spirochaetota* | *Spirochaetia* | *Spirochaetales* | *Spirochaetaceae* | NA | 3 |
|  | NA | NA | NA | NA | NA | 1 |

**Table S5.** Average relative abundance and prevalence of the 40 most abundant taxa for each *Culex* species/form.

| Phylum | Family | Genus | *Cx. pipiens pipiens* Abundance | *Cx. pipiens pipiens* Prevalence | *Cx. pipiens molestus* Abundance | *Cx. pipiens molestus* Prevalence | *Cx. pipiens*  *pipiens / molestus* hybrid Abundance | *Cx. pipiens pipiens / molestu*s hybrid Prevalence | *Cx. quinquefasciatus* Abundance | *Cx. quinquefasciatus* Prevalence | *Cx. restuans* Abundance | *Cx. restuans* Prevalence |
| --- | --- | --- | --- | --- | --- | --- | --- | --- | --- | --- | --- | --- |
| *Actinobacteriota* | *Corynebacteriaceae* | *Corynebacterium* | 0.172 | 5.833 | 0 | 4.348 | 0 | 0 | 0.400 | 26.667 | 0.722 | 3.937 |
|  | *Geodermatophilaceae* | *Blastococcus* | 0.020 | 0.833 | 0 | 0 | 0 | 0 | 0 | 0 | 0.660 | 0.787 |
|  | *Propionibacteriaceae* | *Cutibacterium* | 0.078 | 13.333 | 0 | 4.348 | 0.007 | 16.667 | 0.094 | 20 | 0.717 | 7.087 |
|  | *Rubrobacteriaceae* | *Rubrobacter* | 0 | 0.833 | 0.649 | 4.348 | 0 | 0 | 0 | 0 | 0 | 0 |
|  | *Solirubrobacteraceae* | *Patulibacter* | 0.394 | 0.833 | 0 | 0 | 0 | 0 | 0.199 | 6.667 | 0 | 0 |
| *Bacteroidota* | *Weeksellaceae* | *Chryseobacterium* | 0 | 0.833 | 0 | 4.348 | 0 | 2.778 | 0.552 | 6.667 | 0.492 | 3.937 |
| *Cyanobacteria* | *Desertifilaceae* | *Oscillatoria* | 0.447 | 66.667 | 0.068 | 60.870 | 0.296 | 86.111 | 0.209 | 80 | 17.323 | 78.740 |
| *Firmicutes* | *Family XI* | *Anaerococcus* | 0.832 | 2.500 | 0 | 0 | 0 | 2.778 | 0.002 | 6.667 | 0.537 | 0.787 |
|  | *Gemellaceae* | *Gemella* | 0.036 | 3.333 | 0 | 0 | 0.414 | 5.556 | 0 | 0 | 0.726 | 2.362 |
|  | *Ruminococcaceae* | *Faecalibacterium* | 0 | 2.500 | 0 | 0 | 0 | 2.778 | 0 | 0 | 0.778 | 0.787 |
|  | *Staphylococcaceae* | *Staphylococcus* | 0.050 | 5.833 | 0 | 8.696 | 0 | 8.333 | 0.532 | 20 | 1.703 | 7.874 |
|  | *Streptococcaceae* | *Streptococcus* | 0.062 | 5.833 | 0 | 0 | 0.342 | 5.556 | 0 | 6.667 | 0.970 | 11.811 |
|  | *Vagococcaceae* | *Vagococcus* | 0.186 | 5.000 | 0 | 4.348 | 0 | 2.778 | 0 | 0 | 0.971 | 8.661 |
| *Proteobacteria* | *Acetobacteraceae* | *Gluconobacter* | 0.474 | 3.333 | 0 | 0 | 0 | 0 | 1.693 | 6.667 | 0.060 | 1.575 |
|  | *Aeromonadaceae* | *Aeromonas* | 0.147 | 25 | 0.322 | 26.087 | 0.252 | 22.222 | 0.001 | 13.333 | 2.081 | 18.898 |
|  | ***Anaplasmataceae*** | ***Wolbachia*** | **80.943** | **100** | **90.357** | **100** | **90.384** | **100** | **84.219** | **100** | **7.966** | **100** |
|  | *Burkholderiaceae* | *Ralstonia* | 0 | 0 | 0 | 0 | 0 | 0 | 0 | 0 | 1.411 | 1.575 |
|  | *Comamonadaceae* | *Acidovorax* | 0 | 0 | 0 | 0 | 0 | 0 | 0 | 0 | 0.757 | 0.787 |
|  |  | *Comamonas* | 0.001 | 3.333 | 0.782 | 8.696 | 0 | 5.556 | 0 | 0 | 1.048 | 8.661 |
|  |  | *Delftia* | 0.360 | 3.333 | 0 | 4.348 | 0.059 | 2.778 | 0 | 0 | 0.759 | 3.937 |
|  |  | *Pelomonas* | 0.459 | 17.500 | 0.024 | 13.043 | 0.004 | 13.889 | 0 | 6.667 | 1.515 | 18.110 |
|  |  | *Variovorax* | 0 | 0 | 0 | 0 | 0 | 0 | 0 | 0 | 0.863 | 1.575 |
|  | *Enterobacteriaceae* | *Enterobacter* | 0.352 | 7.500 | 0 | 4.348 | 0 | 8.333 | 0.001 | 20 | 0.832 | 11.024 |
|  |  | *Escherichia-Shigella* | 2.382 | 43.333 | 1.334 | 39.130 | 0.720 | 66.667 | 0.148 | 46.667 | 5.621 | 40.157 |
|  | *Erwiniaceae* | *Erwinia* | 0.623 | 29.167 | 0.611 | 21.739 | 0.043 | 33.333 | 6.266 | 33.333 | 3.129 | 29.134 |
|  |  | *Pantoea* | 0.217 | 20.833 | 0.080 | 17.391 | 0.027 | 13.889 | 0.002 | 20 | 1.335 | 23.622 |
|  | *Halomonadaceae* | *Zymobacter* | 0.381 | 53.333 | 0.001 | 52.174 | 0.643 | 66.667 | 0.001 | 46.667 | 1.269 | 22.835 |
|  | *Moraxellaceae* | *Acinetobacter* | 0.762 | 19.167 | 0 | 13.043 | 0.037 | 16.667 | 0.074 | 20 | 1.001 | 16.535 |
|  |  | *Providencia* | 2.854 | 70 | 1.805 | 60.870 | 0.002 | 69.444 | 1.712 | 40 | 0.017 | 47.244 |
|  | *Pasteurellaceae* | *Haemophilus* | 0 | 1.667 | 0 | 0 | 0.046 | 2.778 | 0 | 0 | 1.422 | 5.512 |
|  | *Pectobacteriaceae* | *Lonsdalea* | 1.419 | 23.333 | 0.001 | 26.087 | 0.001 | 22.222 | 0.376 | 33.333 | 1.643 | 25.984 |
|  | *Pseudomonadaceae* | *Pseudomonas* | 1.120 | 92.500 | 1.664 | 95.652 | 3.159 | 97.222 | 0.642 | 73.333 | 22.073 | 77.165 |
|  | *Rhodobacteraceae* | *Rubellimicrobium* | 0 | 0.833 | 0.514 | 4.348 | 0.046 | 2.778 | 0 | 0 | 0.283 | 0.787 |
|  | *Rickettsiaceae* | *Ac37b* | 0 | 0.833 | 0.227 | 8.696 | 0.249 | 5.556 | 1.158 | 6.667 | 0 | 0 |
|  | *Sphingomonadaceae* | *Ellin6055* | 0.075 | 0.833 | 0 | 0 | 0 | 0 | 0 | 0 | 0.698 | 0.787 |
|  |  | *Sphingomonas* | 0.314 | 60 | 0.303 | 65.217 | 1.351 | 63.889 | 0.001 | 40 | 6.782 | 36.220 |
|  | *Thorselliaceae* | *Coetzeea* | 1.324 | 25.833 | 0.009 | 4.348 | 0 | 25.000 | 0 | 13.333 | 0.001 | 14.961 |
|  | *Xanthomonadaceae* | *Stenotrophomonas* | 0.240 | 3.333 | 0 | 0 | 0 | 0 | 0 | 0 | 1.346 | 1.575 |
|  | *Yersiniaceae* | *Gibbsiella* | 0.088 | 15.833 | 0 | 8.696 | 0.751 | 11.111 | 0.001 | 20 | 0.001 | 18.110 |
|  |  | *Serratia* | 0.276 | 38.333 | 0.001 | 30.435 | 0.168 | 47.222 | 0.002 | 40 | 2.833 | 37.008 |

**Table S6. ANCOM-BC2 results of the 9 differentially abundant genera between *Cx. restuans* and *Cx. pipiens*.**

| Genus | LFC *Cx. pipiens* v. *Cx. restuans* | p-adj | Enriched in |
| --- | --- | --- | --- |
| *Cutibacterium* | 1.722 | < 0.001 | *Cx. restuans* |
| *Wolbachia* | -7.963 | < 0.001 | *Cx. pipiens* |
| *Sphingomonas* | 1.247 | 0.024 | *Cx. restuans* |
| *Escherichia-Shigella* | 1.278 | 0.022 | *Cx. restuans* |
| *Kluyvera* | 1.166 | < 0.001 | *Cx. restuans* |
| *Gibbsiella* | -3.182 | < 0.001 | *Cx. pipiens* |
| *Acinetobacter* | 1.541 | < 0.001 | *Cx. restuans* |
| *Pseudomonas* | 2.817 | < 0.001 | *Cx. restuans* |
| Unknown | 1.401 | 0.024 | *Cx. restuans* |

**Table S7. Significant pairwise ANCOM-BC2 results of the 2 differentially abundant taxa between *Cx. pipiens* forms.**

| Genus | Comparison | LFC | p adj. | Interpretation |
| --- | --- | --- | --- | --- |
| *Erwinia* | *Cx. pipiens pipiens vs. Cx.*  *pipiens molestus* | -2.121 | 0.002 | *Cx.pipiens molestus > Cx. pipiens pipiens* |
| *Erwinia* | *Cx. pipiens pipiens / molestus hybrid vs. Cx. pipiens molestus* | -3.355 | < 0.001 | *Cx. pipiens molestus > Cx. pipiens pipiens / molestus hybrid* |
| *Erwinia* | *Cx. pipiens quinquefasciatus > Cx. pipiens pipiens* | 4.331 | < 0.001 | *Cx. pipiens quinquefasciatus > Cx. pipiens pipiens* |
| *Erwinia* | *Cx. pipiens quinquefasciatus vs. Cx. pipiens pipiens / molestus hybrid.* | 5.566 | < 0.001 | *Cx. pipiens quinquefasciatus > Cx. pipiens pipiens / molestus hybrid* |
| *Lonsdalea* | *Cx. pipiens pipiens vs. Cx. pipiens molestus* | 1.652 | 0.033 | *Cx. pipiens pipiens > Cx. pipiens molestus* |
| *Lonsdalea* | *Cx. pipiens quinquefasciatus vs. Cx. pipiens molestus* | 3.444 | 0.009 | *Cx. pipiens quinquefasciatus > Cx. pipiens molestus* |
| *Lonsdalea* | *Cx. pipiens quinquefasciatus vs. Cx. pipiens pipiens / molestus hybrid.* | 3.005 | 0.015 | *Cx. pipiens quinquefasciatus > Cx. pipiens pipiens / molestus hybrid* |

**Table S8. Significant pairwise ANCOM-BC2 results of the 14 differentially abundant taxa between sampling regions.**

| Genus | LFC MW vs. CU | p-adj. MW vs. CU | LFC NS vs. CU | p-adj. NS vs. CU | LFC NS vs. MW | p-adj. NS vs. MW | Interpretation |
| --- | --- | --- | --- | --- | --- | --- | --- |
| *Cutibacterium* | - | - | -1.945 | < 0.001 | - | - | CU > NS |
| *Allobacillus* | - | - | -1.012 | 0.007 | - | - | CU > NS |
| *Sphingomonas* | - | - | 1.969 | < 0.001 | - | - | NS > CU |
| *Pelomonas* | - | - | 2.029 | < 0.001 | 2.876 | < 0.001 | NS > CU = MW |
| *Aeromonas* | 1.616 | 0.019 | - | - | - | - | MW > CU |
| *Escherichia-Shigella* | - | - | - | - | -1.719 | 0.028 | MW > NS |
| *Kluyvera* | 1.186 | 0.042 | -0.919 | 0.015 | -2.105 | 0.001 | MW > CU > NS |
| *Erwinia* | - | - | -1.243 | 0.009 | -1.689 | 0.019 | MW = CU > NS |
| *Providencia* | - | - | -1.703 | < 0.001 | - | - | CU > NS |
| *Lonsdalea* | - | - | -1.054 | 0.009 | -2.003 | 0.002 | CU = MW > NS |
| *Coetzeea* | 1.486 | 0.011 | 1.291 | < 0.001 | - | - | MW = NS > CU |
| *Gibbsiella* | 3.638 | < 0.001 | 0.947 | 0.006 | -2.691 | < 0.001 | MW > NS > CU |
| *Zymobacter* | 1.541 | 0.009 | - | - | -1.774 | 0.006 | MW > CU = NS |
| *Acinetobacter* | -1.848 | 0.005 | -3.175 | < 0.001 | -1.327 | 0.032 | CU > MW > NS |

**Table S9. Significant pairwise ANCOM-BC2 results of the 2 differentially abundant taxa between sampling months.**

| Genus | Comparison | LFC | p-adj. | Interpretation |
| --- | --- | --- | --- | --- |
| Pelomonas | June vs. May | 4.019 | 0.017 | June > May |
| Gibbsiella | July vs. May | -4.341 | 0.011 | May > July |
| Gibbsiella | August vs. May | -4.079 | 0.021 | May > August |
| Gibbsiella | July vs. June | -4.826 | 0.003 | June > July |
| Gibbsiella | August vs. June | -4.563 | 0.007 | June > August |
